# Characterizing Surgical and Radiotherapy Outcomes in Non-metastatic High-Risk Prostate Cancer: A Systematic Review and Meta-Analysis

**DOI:** 10.7759/cureus.17400

**Published:** 2021-08-23

**Authors:** David E Guy, Hanbo Chen, R Gabriel Boldt, Joseph Chin, George Rodrigues

**Affiliations:** 1 Radiation Oncology, London Health Sciences Centre, London, CAN; 2 Urology, London Health Sciences Centre, London, CAN; 3 Medicine, Schulich School of Medicine & Dentistry at Western University, London, CAN

**Keywords:** comparative effectiveness research, prostate cancer, radiation oncology, radical prostatectomy, systematic review, meta-analysis

## Abstract

Background

Identifying the optimal management of high-risk non-metastatic prostate cancer (PCa) is an important public health concern, given the large burden of this disease. We performed a meta-analysis of studies comparing PCa-specific mortality (CSM) among men diagnosed with high-risk non-metastatic PCa who were treated with primary radiotherapy (RT) and radical prostatectomy (RP).

Methods

Medline and Embase were searched for articles between January 1, 2005, and February 11, 2020. After title and abstract screening, two authors independently reviewed full-text articles for inclusion. Data were abstracted, and a modified version of the Newcastle-Ottawa Scale, involving a comprehensive list of confounding variables, was used to assess the risk of bias.

Results

Fifteen studies involving 131,392 patients were included. No difference in adjusted CSM in RT relative to RP was shown (hazard ratio, 1.02 [95% confidence interval: 0.84, 1.25]). Increased CSM was found in a subgroup analysis comparing external beam radiation therapy (EBRT) with RP (1.35 [1.10, 1.68]), whereas EBRT combined with brachytherapy (BT) versus RP showed lower CSM (0.68 [0.48, 0.95]). All studies demonstrated a high risk of bias as none fully adjusted for all confounding variables.

Conclusion

We found no difference in CSM between men diagnosed with non-metastatic high-risk PCa and treated with RP or RT; however, this is likely explained by increased CSM in men treated with EBRT and decreased CSM in men treated with EBRT + BT studies relative to RP. High risk of bias in all studies identifies the need for better data collection and confounding control in the PCa research.

## Introduction

Prostate cancer (PCa) was the second most frequently diagnosed cancer and the fifth leading cause of cancer death worldwide as of 2018 [1]. High-risk PCa - as defined by a clinical stage ≥ T3, Gleason score of 8-10, or prostate-specific antigen (PSA) > 20 ng/ml at the time of diagnosis [2] - accounts for approximately one-quarter of all PCa diagnoses but was responsible for a disproportionately larger share of PCa-specific mortality (CSM) [3]. Optimal selection and sequencing of therapy for high-risk non-metastatic PCa, such as the choice between radical prostatectomy (RP) and radical radiotherapy (RT), remain an area of intense academic and clinical debate [4]. Unfortunately, no randomized controlled trials (RCTs) on this topic have been completed due to the low patient and provider equipoise surrounding RP and RT, especially in North America [5,6]. As such, investigations comparing RP and RT outcomes have mostly been performed using non-randomized data. In the absence of RCTs, meta-analyses that summarize high-quality non-randomized data can inform treatment decisions for physicians and policymakers.

Previous meta-analyses that have compared mortality outcomes between patients diagnosed with PCa and treated with RP or RT involved studies that compared older treatment approaches, which greatly differ from current standards of care [7]. Publications included in these meta-analyses have since been updated to include longer follow-up periods of more contemporary RT approaches such as dose-escalation protocols for external beam radiation therapy (EBRT), use of brachytherapy boost (BT), and adjuvant androgen deprivation therapy (ADT) [8-11], which may lead to better oncological outcomes for men diagnosed with high-risk non-metastatic PCa [12-14]. Although a more recent meta-analysis has been conducted [15], numerous errors were made, limiting the utility of the aggregated effect estimates for use in clinical practice. For instance, multiple effect estimates were generated from overlapping data [9,16-22] leading to some patient data overinfluencing aggregate effect estimates as well as the inclusion of a study investigating low-risk PCa [10]. Moreover, the authors aggregated studies involving patients diagnosed with non-metastatic and nodal metastatic high-risk PCa [19], which have heterogeneous disease trajectories and ultimately call for different management approaches that are not comparable [23].

The objective of this study was to compare the relative rates of CSM and ACM between men diagnosed with high-risk non-metastatic PCa and treated with RP or RT as their primary treatment modality.

## Materials and methods

Research question

The primary and secondary objectives of the study were to summarize the relative CSM and ACM, respectively, of patients diagnosed with non-metastatic high-risk PCa treated primarily with either RP or RT.

Protocol and search strategy

The systematic review was conducted in accordance with the Preferred Reporting Items for Systematic Reviews and Meta-Analyses (PRISMA) guidelines [24]. The review protocol has been registered in the International Prospective Register of Systematic Reviews (PROSPERO) (registration number: CRD42020150710). The search strategy is provided in Appendix 1. Studies were included in our analysis if they were published between January 1, 2005, and February 11, 2020, to limit attention to analyses of more contemporary treatment periods. Only full-text articles published in English in a peer-reviewed journal were considered.

We included only cohort studies in our review since case-control studies could not typically evaluate hazard ratios. Furthermore, previous RCTs were excluded due to insufficient numbers of men diagnosed with non-metastatic high-risk PCa to form valid inferences [25]. Editorials, letters to the editor, commentaries, guidelines, and review articles were also excluded.

We included studies that reported on men of any age diagnosed with non-metastatic high-risk PCa, according to the National Comprehensive Cancer Network (clinical stage ≥ T3, Gleason score of 8-10, or prostate-specific antigen > 20 ng/ml) [2] or D’Amico criteria (clinical stage ≥ T2c, Gleason score 8-10, or prostate-specific antigen > 20 ng/ml) who were treated with either primary RP or RT [26]. All common forms of RP (e.g., open retropubic, laparoscopic, and robotic) and RT (e.g., conformal external beam, intensity-modulated, brachytherapy, or combination of radiotherapy modalities with curative intent) were considered. Studies assessing adjuvant or salvage therapies as the primary objective were excluded. We included only studies that provided a hazard ratio for CSM or ACM, both adjusted for confounding. Studies reporting on surrogate outcome measures such as biochemical progression were excluded since definitions for RP and RT differ.

Article review

**Figure 1:**
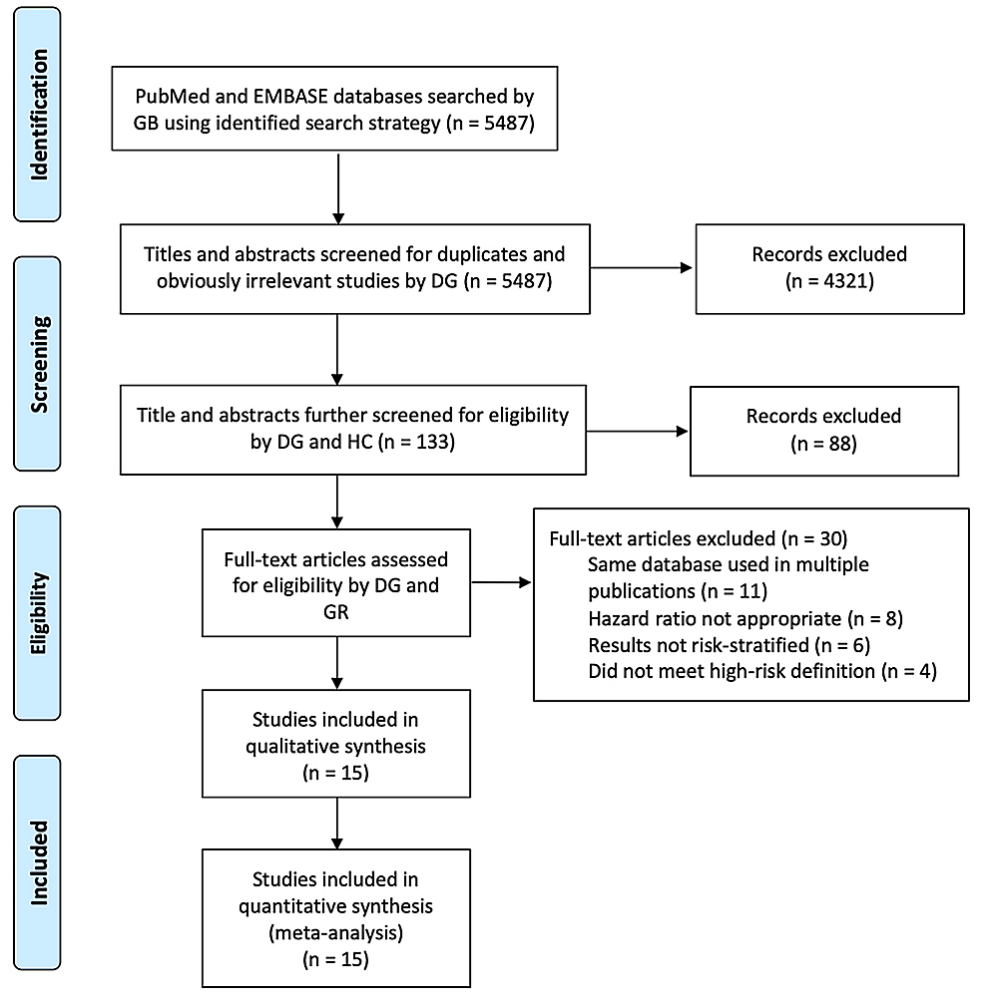
PRISMA flow diagram outlining search strategy and the final list of included and excluded studies PRISMA, Preferred Reporting Items for Systematic Reviews and Meta-Analyses.

The first phase of the project involved title and abstract review by DG to discard non-relevant citations and duplications. Full-text reviews of the remaining studies were examined in the second phase by DG and HC to determine eligibility for inclusion based on pre-determined criteria. Afterward, DG and HC independently reviewed the records, and GBR settled discrepancies on the inclusion/exclusion of certain records. When more than one publication existed using the same patient population, the most relevant, updated, and complete publication was selected. A diagram describing the study flow is outlined in Figure 1.

Data extraction and risk of bias assessment

A data extraction form was completed for each study as outlined in Appendix 2. We used a modified Newcastle-Ottawa Scale to include a comprehensive list of items identifying confounding variables (see Appendix 3). Confounding variables included those relating to tumor characteristics (baseline PSA, Gleason score, and clinical stage), age, comorbidity status, year of diagnosis or treatment, study center (if multiple), and at least one demographic characteristic (e.g., education, income, rural or urban residence). This list was reviewed and approved by both a radiation oncologist (GR) and an uro-oncologist (JC).

Publication bias

We assessed publication bias using funnel plots and the Egger test. Hazard ratios from included studies were plotted as a function of their standard error in relation to the aggregate effect estimate generated through random-effects models. Residual values were also estimated using mixed-effects models to account for heterogeneity due to moderator variables (RT approach for CSM and ACM, and age for ACM) in order to improve interpretation of funnel plots for the assessment of publication bias.

Assessment of heterogeneity

The Q-test was performed to identify significant heterogeneity in treatment effect estimates, using the DerSimonian-Laird method, and quantified through the I2 statistic [27].

Statistical analysis

General study information, PCa treatment and endpoint information, and methodological information were categorized into tables using frequency or proportions for categorical variables, medians or means for continuous variables, and descriptive terms for other variables where appropriate.

The meta-analysis was performed in R statistical software (x64, version 3.3.2; R Foundation for Statistical Computing, Vienna, Austria) with the “metafor” package (version 1.9-9) [28]. The primary meta-analysis comparing CSM between RP and RT was carried out using inverse variance-weighted random-effects models. We then performed a series of univariable meta-regression to explore sources of heterogeneity. Input variables included treatment era (examined as a binary variable with values of 1 and 0 for values above and below the median year of diagnosis, respectively), approach to RT (EBRT with or without brachytherapy boost), length of follow-up (examined as a binary variable with values of 1 and 0 for values above and below the median, respectively), geographical location (the United States versus other), and age (examined as a binary variable with values of 1 and 0 for values above and below the median, respectively). Insufficient data were available to explore the effect of RT dose, RP approach (i.e., open, laparoscopic, robotic), the proportion receiving systemic therapy (i.e., ADT, chemotherapy, and adjuvant RT), and type of EBRT (i.e., 3D conformal, IMRT, etc.). All statistical tests were two-sided with significance levels of <0.05.

## Results

Fifteen studies involving 131,932 total patients were identified for inclusion. The article selection flowchart is outlined in Figure 1.

Study characteristics

Table 1 shows the characteristics of individual studies. Four studies compared treatment groups from a single institution, another four studies compared groups from different institutions, another five studies used national registries to compare treatment groups, and two studies made comparisons across multiple institutions. Patient characteristics varied across studies due to variations in inclusion and exclusion criteria. In general, RT patients were older, had a greater number of comorbidities, and had poorer prognostic characteristics. Median follow-up varied substantially between studies and treatment groups. Treatment details were scarcely reported for the RP group, while details regarding RT dose, the proportion receiving ADT, and whether EBRT was performed in conjunction with BT were provided in most studies.

**Table 1:** General characteristics of included studies RP = Radical prostatectomy; RT = radiation therapy; Gy = Gray; EBRT = external beam radiation therapy; BT = brachytherapy; SEER = Surveillance, Epidemiology, and End Results Program; ADT = androgen deprivation therapy; I125 = Iodine-125; Pd103 = Palladium-103; Cs131 = Cesium-131.

Author	Year	Treatment comparison	Data source (study interval)	Median follow-up duration (RP/RT), months	RP (n)	RT (n)	Median age (RP/RT), years	Median RT dose (Gy)	Adjuvant therapy
Yin et al. [11]	2019	EBRT + BT ± ADT v RP	SEER 21 (2004, 2015)	58/87	59540	355	63.8/66.1	na	ADT: RT: "majority", RP: na
		EBRT ± ADT v RP		/62		2638	/69.4		
Jayadevappa et al. [20]	2019	EBRT + BT ± ADT v RP	SEER-Medicare (1996, 2003)	≥120	677	4141	71.7/73.1	na	Not reported
		EBRT + ADT v RP				1478	/75.5		ADT: RT: 100%, RP: na
Gunnarsson et al. [22]	2019	EBRT ± BT ± ADT v RP + RT + ADT	Kalmar County Hospital, Sweden (RP); the National Prostate Cancer Register (RT) (1995, 2010)	na	153	702	65/65	EBRT ≤78 or EBRT 20 + BT 50	ADT: RT: "preferred" RP: 100%, aRT: 64%
Cano-Velasco et al. [29]	2019	EBRT + ADT v RP + ADT	Hospital General Universitario Gregorio Maran ~ón, Madrid, Spain (1996, 2008)	152/97	145	141	65/71	EBRT 74	ADT: RT: 100%, RP: 100%
Tilki et al. [30]	2018	EBRT + BT + ADT v RP	Chicago Prostate Cancer Centre (RT); Martini-Klinik Prostate Cancer Center (RP) (1992, 2013)	58.7/66.1	372	80	66.4/70.3	EBRT 45 BT (I125, Pd103 and Cs131) 108/90/100	ADT: RT: 100%, RP: 0%, aRT: 0%
		v RP + ADT		46.4/	88		66.6/		RP: 100%, aRT: 0%
		v RP + aRT		58.6/	49		66/		RP: 0%, aRT: 100%
		v RP + ADT + aRT		57.4/	50		66.4/		RP: 100%, aRT: 100%
Ennis et al. [31]	2018	EBRT + BT ± ADT v RP	National Cancer Database (2004, 2013)	36.3	24688	15435	62.6/67.2	na	ADT: RT: na, RP: na
		EBRT + ADT v RP				2642	/69.7		ADT: RT: 100%, RP: na
Robinson et al. [9]	2018	EBRT ± BT ± ADT v RP	Swedish National Prostate Cancer Registry (1998, 2012)	75.6/70.8	3761	6462	63.1/67	na	Not reported
Ciezki et al. [32]	2017	EBRT v RP	Cleveland Clinic (1996, 2012)	55.6/94.6	1308	734	62/68.5	(52%) at 78 (2 Gy fraction) & (48%) at 70 (2.5 Gy fraction)	ADT: RT: 93%, RP: 19%
		EBRT + BT v RP		/48.9		515	/70		ADT: RT: 53%, RP: 19%
Kishan et al. [33]	2017	EBRT v RP	Multi-institutional (12 centers) (2000, 2013)	50.4/61.2	639	734	61.2/68	EBRT 74.3	ADT: RT: 89.5%, RP: 39%, aRT: 34%
		EBRT + BT v RP		50.4/75.6		436	/68		ADT: RT: 92.4%, RP: 39%, aRT: 34%
Greenberg et al. [34]	2015	EBRT + ADT v RP	Anglia Cancer Network, UK (2000, 2010)	na/na	na	na	na/na	na	ADT: RT: 88.2%, RP: na
Lee et al. [35]	2014	EBRT ± ADT v RP	Severance Hospital, Seoul, Korea (1990, 2009)	74/85.5	251	125	67.5/68.6	EBRT (range) 74-79	Not reported
Yamamoto et al. [36]	2014	EBRT ± ADT v RP	Cancer Institute Hospital in Tokyo, Japan (1994, 2005)	93/85	112	119	67/72	EBRT 70	ADT: RT: 95.8%, RP: 76.8%
Westover et al. [37]	2012	EBRT + BT + ADT v RP	Duke University (RP) (1988, 2008); Chicago Prostate Cancer Centre 21st Century Oncology Establishment (RT) (1991, 2005)	91.2/43.2	285	372	65/70	EBRT 45 BT I125/Pd103 108/90	ADT: RT: 100%, RP: 0%
Kibel et al. [38]	2012	EBRT v RP	Barnes-Jewish Hospital and Cleveland Clinic (1995, 2005)	(59 to 72)/(70 to 74)	525	676	60.4/69.4	EBRT (median) 74 to 78 BT na	ADT: RT: 82%, RP: na
		EBRT + BT v RP		(59 to 72)/(51 to 70)		33	/68.4		
Boorjian et al. [39]	2011	EBRT v RP	Mayo Clinic Prostatectomy Registry (RP) and the Fox Chase Cancer Centre (RT) (1988, 2004)	122.4/87.6	1238	344	66/69.3	EBRT 72	ADT: RT: 0%, RP: 40.6%
		EBRT + ADT v RP		/72		265	/68.8		ADT: RT: 100%, RP: 40.6%

Risk of bias assessment

**Figure 2:**
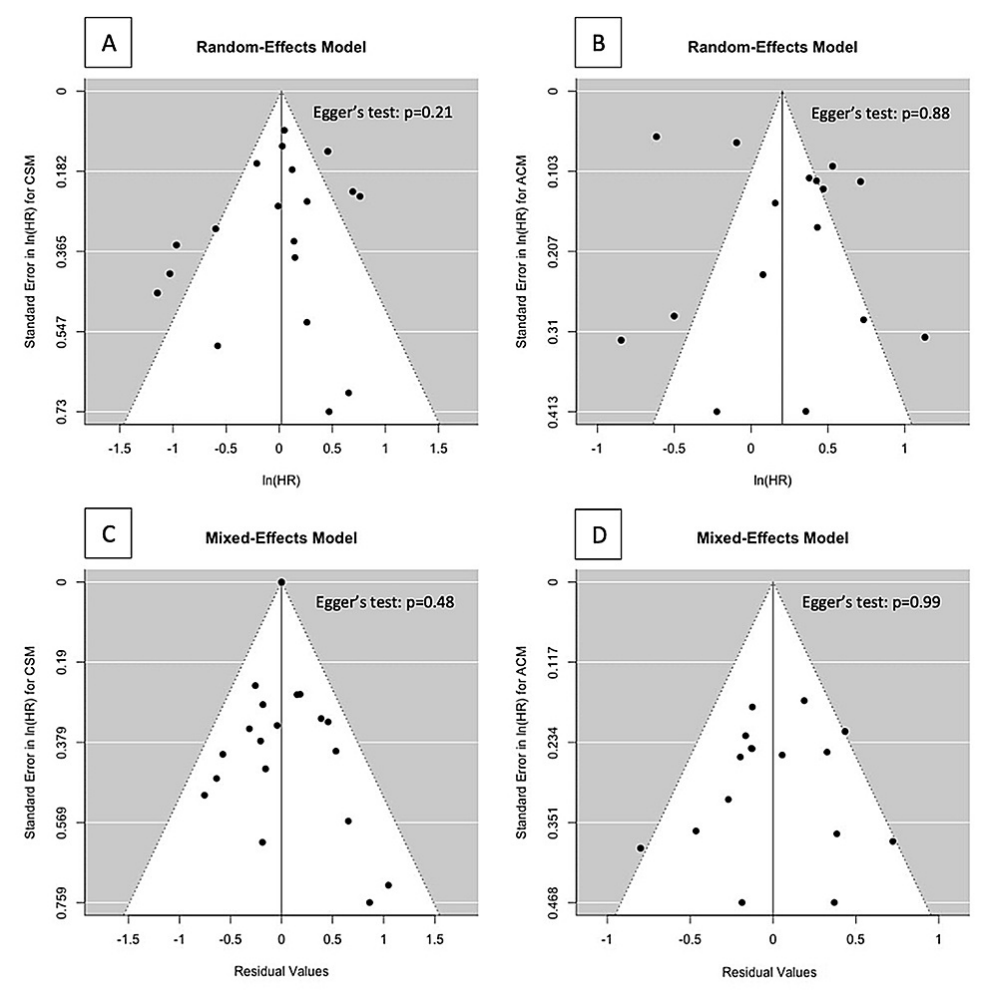
Funnel plots of meta-analysis for (A) prostate cancer-specific mortality, (B) all-cause mortality using random-effects models, (C) CSM adjusted for the receipt of BT, and (D) for all-cause mortality adjusted for receipt of BT and age using mixed-effects models HR = Hazard ratio; CSM = PCa-specific mortality; BT = brachytherapy boost.

The overall risk of bias was high for all studies (Table 2) as none adjusted for all potential confounders. Most studies had a low risk of bias for the ‘selection’ section other than those comparing the treatment groups from tertiary centers. The ‘comparability’ section varied due to variation in covariate control. All studies controlled for age; most studies provided adequate control for tumor characteristics (i.e., PSA, clinical stage, and Gleason score) (14/15), while fewer studies controlled for comorbidities (8/15), demographic characteristics (5/15), and study center (8/15). Finally, most studies did not have a sufficient median follow-up, leading to a score of 2/3 for the ‘outcome’ section for 13/15 studies. There was no indication of publication bias. The Egger test for publication bias was not statistically significant (p = 0.21 for CSM and 0.88 for ACM; Figure 2).

**Table 2:** Modified Newcastle-Ottawa Scale for risk of bias assessment of studies included in the meta-analysis RP = Radical prostatectomy; RT = radiation therapy; cT = clinical stage; GS = Gleason score; PSA = prostate-specific antigen.

Study information	Selection													
Author (Year)	Representativeness of the exposed cohort (RT)				Representativeness of the non-exposed cohort (RP)		Ascertainment of exposure			Demonstration that outcome of interest was not present at start				Total
Yin et al. (2019)	1				1		1			1				4
Jayadevappa et al. (2019)	1				1		1			1				4
Gunnarsson et al. (2019)	1				0.5		1			1				3.5
Cano-Velasco et al. (2019)	0.5				0.5		1			1				3
Tilki et al. (2018)	0.5				0.5		1			1				3
Ennis et al. (2018)	1				1		1			1				4
Robinson et al. (2018)	1				1		1			1				4
Ciezki et al. (2017)	0.5				0.5		1			1				3
Kishan et al. (2017)	1				1		1			1				2
Greenberg et al. (2015)	1				1		1			1				4
Lee et al. (2014)	1				1		1			1				4
Yamamoto et al. (2014)	0.5				0.5		1			1				3
Westover et al. (2012)	0.5				0.5		1			1				3
Kibel et al. (2012)	1				1		1			1				4
Boorjian et al. (2011)	0.5				0.5		1			1				4
	Comparability													
Author (Year)	cT	GS	PSA	Age		Comorbidity	Demographic characteristic		Year of diagnosis or treatment			Study center et al. (if applicable)		Total
Yin et al. (2019)	1	1	1	1		0	1		1			1		3.5
Jayadevappa et al. (2019)	0	0	0	1		1	1		1			1		2.5
Gunnarsson et al. (2019)	0.5	0.5	0.5	1		0	1		0			0		1.75
Cano-Velasco et al. (2019)	1	1	1	1		0	0		0			1		2.5
Tilki et al. (2018)	1	1	1	1		1	1		0			0		3
Ennis et al. (2018)	1	1	1	1		1	1		1			0		3.5
Robinson et al. (2018)	1	1	1	1		1	1		1			1		3.5
Ciezki et al. (2017)	1	1	1	1		0	0		0			1		2.5
Kishan et al. (2017)	1	1	1	1		0	0		0			1		2.5
Greenberg et al. (2015)	1	1	1	1		0	0		0			0		2
Lee et al. (2014)	1	1	1	1		1	1		0			1		3.5
Yamamoto et al. (2014)	1	1	1	1		1	0		0			1		3
Westover et al. (2012)	1	1	1	1		1	1		0			0		3
Kibel et al. (2012)	1	1	1	1		1	0		1			0		3
Boorjian et al. (2011)	1	1	1	1		0	1		0			0		2.5
	Outcome													
Author (Year)	Ascertainment of outcome					Adequate cohort follow-up intensity		Sufficient follow-up duration			Total		Risk of bias	
Yin et al. (2019)	1					1		0			2		High	
Jayadevappa et al. (2019)	1					1		0			2		High	
Gunnarsson et al. (2019)	1					1		0			2		High	
Cano-Velasco et al. (2019)	0					1		1			2		High	
Tilki et al. (2018)	1					1		0			2		High	
Ennis et al. (2018)	1					1		0			2		High	
Robinson et al. (2018)	1					1		0			2		High	
Ciezki et al. (2017)	1					1		0			2		High	
Kishan et al. (2017)	1					1		0			2		High	
Greenberg et al. (2015)	1					1		0			2		High	
Lee et al. (2014)	1					0		0			1		High	
Yamamoto et al. (2014)	1					1		0			2		High	
Westover et al. (2012)	1					1		0			2		High	
Kibel et al. (2012)	1					1		0			2		High	
Boorjian et al. (2011)	1					1		1			3		High	

Prostate cancer-specific mortality

**Figure 3:**
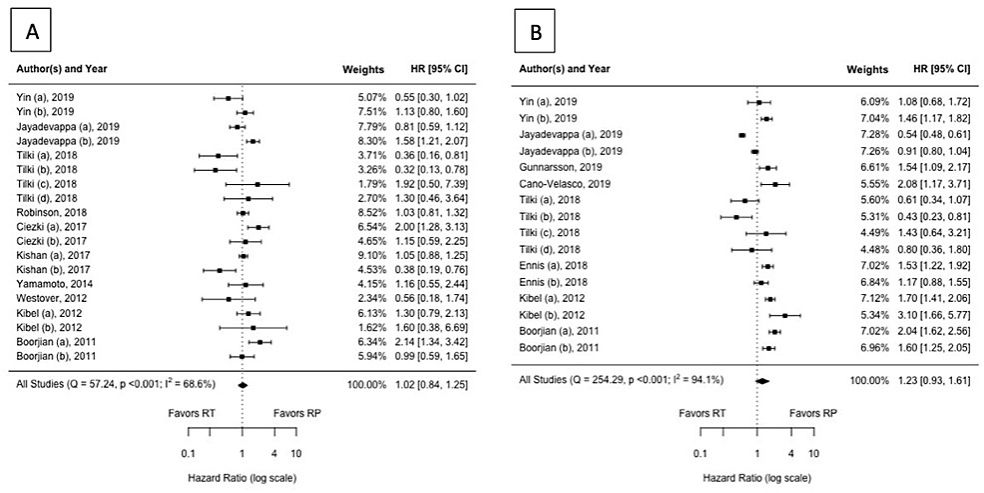
Forest plot assessing the risk of (A) prostate cancer-specific mortality and (B) all-cause mortality following radiotherapy and surgery for prostate cancer HR = Hazard ratio; CI = confidence interval; RT = radiation therapy; RP = radical prostatectomy.

Ten studies with 88,026 patients were included in the primary meta-analysis for CSM. The resulting adjusted hazard ratio [95% confidence interval] was 1.02 [0.84, 1.25] with substantial heterogeneity (I^2 ^= 69%) as shown in Figure 3A. Subgroup analysis revealed a significant effect by the RT approach (p < 0.0001). Specifically, CSM was increased among EBRT ± ADT compared to RP (1.35 [1.10, 1.68]; p = 0.0048) but decreased among EBRT + BT ± ADT compared to RP (0.68 [0.48, 0.95]; p = 0.024) (Table 3). This was also associated with decreased, though still substantial, heterogeneity (I^2 ^= 59% and 47%, respectively). The remaining subgroup analyses did not differ notably from the primary analysis.

**Table 3:** Subgroup analyses assessing the risk of prostate cancer-specific mortality and all-cause mortality following radiotherapy and surgery for prostate cancer N = Number of estimates included in aggregate HR (this might include multiple estimates from unique comparisons from the same publication); HR = hazard ratio; CI = confidence interval; EBRT = external beam radiation therapy; BT = brachytherapy; ADT = androgen deprivation therapy.

	Prostate cancer-specific mortality			Overall mortality		
	N	Adjusted HR (95% CI; p-value)	I^2^	N	Adjusted HR (95% CI; p-value)	I^2^
Radiotherapy modality						
EBRT ± ADT	n = 8	1.35 (1.10, 1.67; p = 0.0048)	59%	n = 7	1.53 (1.18, 1.99; p = 0.0013)	90%
EBRT + BT ± ADT	n = 10	0.68 (0.48, 0.95; p = 0.024)	47%	n = 8	0.93 (0.60, 1.42; p = 0.72)	89%
Treatment Era						
Before 2002.5	n = 12	1.03 (0.75, 1.42; p = 0.84)	69%	n = 12	1.20 (0.84, 1.70; p = 0.32)	95%
After 2002.5	n = 7	1.00 (0.76, 1.30; p = 0.98)	71%	n = 4	1.37 (1.19, 1.58; p < 0.0001)	12%
Age						
≤67.4 years	n = 10	1.04 (0.84, 1.29; p = 0.72)	59%	n = 9	1.52 (1.33, 1.73; p < 0.0001)	42%
>67.4 years	n = 9	0.97 (0.63, 1.47; p = 0.87)	77%	n = 7	0.94 (0.62, 1.43; p = 0.78)	95%
Median follow-up						
≤67.2 months	n = 10	0.80 (0.58, 1.12; p = 0.20)	61%	n = 8	1.16 (0.85, 1.58; p = 0.34)	78%
>67.2 months	n = 9	1.20 (0.93, 1.56; p = 0.16)	72%	n = 7	1.28 (0.83, 1.98; p = 0.27)	97%
Geographic region						
United States	n = 13	1.10 (0.87, 1.38; p = 0.42)	71%	n = 10	1.35 (0.96, 1.88; p = 0.084)	96%
Other	n = 6	0.81 (0.49, 1.32; p = 0.40)	62%	n = 6	1.01 (0.61, 1.66; p = 0.98)	77%

All-cause mortality

Eight studies with 116,975 patients were included in the secondary meta-analysis for ACM. The resulting adjusted HR [95%CI] was 1.23 [0.93, 1.61] with substantial heterogeneity (I^2 ^= 94%) as shown in Figure 3B. Subgroup analysis revealed a significant effect by the RT approach (p = 0.02). Specifically, ACM was increased among EBRT ± ADT compared to RP (1.53 [1.18, 1.99; p = 0.0013]), but there was no significant difference among those treated with EBRT + BT ± ADT relative to RP (0.93 [0.60, 1.42; p = 0.72]) (Table 3). Both subgroup analyses were associated with substantial heterogeneity (I^2^ = 90% and 89%, respectively). Subgroup analysis by median age also revealed a significant effect (p < 0.0001). A significantly higher rate of ACM among RT relative to RP was observed among studies with younger patient groups (1.52 [1.33, 1.73]; p < 0.0001; I^2^ = 42%) compared to those with older patient groups (0.94 [0.62, 1.43]; p = 0.78; I^2^ = 95%) (Table 3). Effect estimates also varied from the main analysis among subgroup analyses of studies performed in the United States (1.35 [0.96, 1.88]) versus other geographic locations (1.01 [0.61, 1.66]).

## Discussion

Our aggregate effect estimates for adjusted CSM showed no statistically significant differences between RP and RT for high-risk non-metastatic PCa patients. Subgroup analysis revealed a significantly increased incidence of CSM among men treated with EBRT ± ADT relative to the RP group and a decreased incidence of CSM among men treated with EBRT + BT ± ADT relative to the RP group. This is consistent with the results from the ASCENDE-RT trial (androgen suppression combined with elective nodal and dose-escalated radiation therapy) wherein an increased incidence of biochemical failure was found among men diagnosed with intermediate- and high-risk non-metastatic PCa and treated with dose-escalation RT protocols using EBRT alone compared with those using combination EBRT + BT (HR [95%CI]: 2.04 [1.25, 3.33]) [13]. Although biochemical failure is not an accepted surrogate and CSM was not significantly different between these groups, the remaining subgroup analyses did not differ from the primary analysis.

Multiple reports indicate that since the early 2000s, the use of BT boost in high-risk patients has declined in the United States [40] and other geographic regions [41]. However, the use of prostate BT boost has increased since the early 2000s in certain European centers and Canada interestingly [42,43]. This discrepancy may be attributable to differences in resident exposure in providing sufficient training opportunities, given the steep learning curve associated with administering BT [44-46] and unfavorable reimbursement relative to EBRT in the United States relative to publicly funded healthcare systems [42,47]. Given the CSM benefit associated with BT boost among high-risk patients reported in RCTs and estimated here, we encourage investment in overcoming the aforementioned obstacles through increasing resident exposure and improving reimbursement models to encourage the use of BT boost.

The HR comparing the relative incidence of CSM between EBRT ± ADT and RP groups was smaller compared to that in a previous meta-analysis performed in 2016 (1.35 [1.10, 1.68] versus 1.83 [1.51-2.22]) [48]. These differences might be explained by more recent changes in treatment approaches including the increasing use of dose-escalation protocols and adjuvant ADT paired with RT [40,41], which have both demonstrated improvements in oncological outcomes, though only the addition of neoadjuvant ADT to RT has demonstrated improvements in CSM [7,12,41].

The analysis of relative ACM between RT and RP also revealed no significant difference between the treatment groups. However, subgroup analysis revealed a significantly increased incidence of ACM among the EBRT ± ADT relative to the RP group, while there was an insignificant decrease in ACM between the EBRT + BT ± ADT and RP groups. In addition to the CSM benefit afforded through RP and EBRT + BT ± ADT relative to EBRT ± ADT, differences in cardiopulmonary health requirements before undergoing general anesthetic that is required for RP and BT and lack of control for comorbidities in many of the included studies might contribute to the observed differences. Studies conducted among younger age groups demonstrated an increased incidence of ACM in the RT relative to the RP group. Finally, a tendency toward increased incidence of ACM in the RT relative to the RP group was also noted among studies conducted only in the United States. However, this is likely explained by the greater proportion of comparisons with RP involving EBRT ± ADT instead of EBRT + BT ± ADT among studies performed in the United States versus other geographic locations.

Overall, the risk of bias was deemed high for all studies due to the partial control of confounding variables. This stands in contrast with a previous meta-analysis performed by Wallis et al. who found a low to moderate risk of bias for all studies included in their meta-analysis comparing the rate of ACM and CSM between patients who underwent RT and RP. Interestingly, four studies used in both analyses indicated perfect comparability between RT and RP groups by Wallis et al., yet some of these studies did not control for study center [37-39], year of diagnosis [35,37,38], or demographic characteristics [38]. Since patients undergoing RT are more likely to be older, have poorer prognostic characteristics, and have sociodemographic characteristics that are associated with poorer CSM and ACM [11,20,29], we anticipate the influence of these unaccounted-for biases to overestimate CSM and ACM in the RT group relative to the RP group. However, the discrepancy in such baseline characteristics appears more prominent among those undergoing EBRT ± ADT rather than EBRT + BT ± ADT wherein patients are more similar to those undergoing RP [11,20]. As such, collecting information on these variables and properly controlling them are crucial when estimating relative treatment effects between groups to more accurately inform treatment decisions.

Our study has certain limitations. There was a high level of heterogeneity in effect estimates. This was substantially reduced through subgroup analyses comparing RP with EBRT ± ADT and EBRT + BT ± ADT, and among comparisons involving younger populations, heterogeneity still remained high and was unaccounted for through additional subgroup analyses. Unfortunately, information surrounding treatment details such as RT dose, type of EBRT (i.e., 3D conformal, IMRT, etc.), use of adjunct therapies, and surgeon experience, which might account for a large proportion of this heterogeneity, was missing in many of the studies.

The aggregated effect estimates provided in this study can be used to inform clinical decisions in conjunction with evidence surrounding quality of life outcomes. Given the relatively small difference in CSM between treatment approaches, other factors such as patient preferences, patient health (i.e., comorbidities), and treatment factors (e.g., operative risk and prostate volume for BT) should be considered when forming treatment decisions. This should occur through a shared decision-making process, involving the patient and providing urologists and radiation oncologists to optimize satisfaction in patient outcomes.

## Conclusions

We identified no significant difference in the relative rate of CSM between patients diagnosed with high-risk non-metastatic PCa and treated with RP relative to RT. However, there was a significant subgroup effect with the use of EBRT + BT ± ADT, highlighting the necessity of differentiating RT with or without BT in future comparative effectiveness studies. The high risk of bias in all studies reviewed emphasizes the need for better control of all potentially confounding variables to provide higher quality non-randomized evidence. This is exceedingly important when RCTs are unlikely to be feasible in this patient population.

## Appendices

Appendix 1: Data extraction items

a) General Study Information

- Title

- Authors

- Publication date

- Study design

· Prospective vs retrospective

- Data source

· National-level databases

· Single-institutional

· Multi-institutional

· Range of calendar years of diagnosis and treatment included

· Geographical location

b) Prostate Cancer, Treatment, and Endpoint Information

- Dates of patient inclusion

- Follow-up duration

- Median age in each group

- Treatment information:

· Number treated in each group

· Approach to radiotherapy (e.g., dose, fractions, duration, 3D, IMRT, brachytherapy, dose-escalation, proton beam, SBRT, combination, etc.)

· Approach to radical prostatectomy (e.g., open-retropubic or perineal, laparoscopic or robotic)

· Use of neoadjuvant or adjuvant hormonal or chemotherapy and duration

- Adjusted HR for prostate cancer-specific mortality and all-cause mortality

Appendix 2: Search strategy

A search strategy was performed by Gabriel Boldt, a clinician librarian, and yielded a total of 5,487 articles between PubMed and EMBASE databases before screening. Search strategies were completed as follows:

PubMed strategy

(radiotherapy[mh] OR radiation therapy[tw] OR radiotherapy[tw] OR surgery[mh] OR prostatectomy[tw] OR surgeries[tw])

AND

prostat*[tw]

AND

surviv*[tw]

AND

(high risk[tw] OR intermediate[tw] OR non-metastatic[tw] OR nonmetastatic[tw] OR localised[tw] OR localized[tw] OR locally[tw] OR local[tw])

NOT

review[pt]

Limits: Human, 2005-2020, English

Results 4325

EMBASE strategy

(radiotherapy.mp. or exp radiotherapy/ or radiation therapy.mp. or surgery.mp. or exp surgery/ or exp prostatectomy/ or prostatectomy.mp. or surgeries.mp.)

and

(prostate tumor/ or prostat*.mp. or exp prostate carcinoma/ or exp prostate cancer/ or exp prostate hypertrophy/)

and

(surviv*.mp. or exp survival/)

and

(high risk or intermediate or non-metastatic or nonmetastatic or localised or localized or locally or local).mp.

limit to (human and English language and exclude Medline journals and yr="2005 -Current")

Results 1162

Appendix 3: Modified Newcastle-Ottawa scale for risk of bias assessment

Items having the potential to bias the relationship between treatment modality (i.e., radical prostatectomy (RP) or radiation therapy (RT)) and outcomes of interest (i.e., cancer-specific or overall survival).

Selection

1. Representativeness of the exposed cohort

a. 1 point for data representing the general population (i.e., in terms of socioeconomic and demographic characteristics)

b. 0 points if data is not representative or indicated (e.g., selected group of users like nurses, volunteers, insured, safety-net hospitals, secondary data from other clinical populations, etc.)

2. Representativeness of the non-exposed cohort

a. 1 point if drawn from the same community as the exposed cohort

b. 0 points if drawn from a different source or not specified

3. Ascertainment of exposure

a. 1 point if obtained from a secure record (e.g., surgical records) or self-report

b. 0 points if no description

4. Demonstration that outcome of interest was not present at the start

a. 1 point if yes

b. 0 points if no

5. Comparability of treatment groups after matching (if applicable) or accounted for the multivariable analysis. Maximum of four points awarded if the following factors are controlled for or not significantly different after matching as indicated by a standardized mean difference >0.10 or p > 0.05:

i. TNM

ii. GS

iii. PSA

iv. Comorbidity status

v. Age

vi. ≥1 year of diagnosis or treatment

vii. ≥1 demographic characteristic (education, income, rural/urban)

viii. Study center (if multiple)

0.5 points were deducted for each variable not included in the model unless tested and shown to have an insignificant influence on the final results.

6. Ascertainment of outcome [1]

a. 1 point if record linkage or blind assessment

b. 0 points if the assessment is not blinded or not reported

7. Adequacy of follow-up of cohorts [2]

a. 1 point if no subjects lost to follow-up or those lost are unlikely to introduce bias (i.e., number lost ≤ 20% or description of those lost suggested no different from those followed)

b. 0 points if follow-up rate < 80% and no description of those lost or if no statement was made

8. Was follow-up long enough for outcomes to occur?

a. 1 point if median follow-up was ≥10 years, as 10-year cancer-specific survival is estimated to be 88% in patients diagnosed with high-risk PCa undergoing multimodal treatment [3].

Thresholds for converting to low, moderate, and high risks of bias:

a. **Low risk of bias:** ≥3 points in selection domain AND 4 points in comparability domain AND ≥2 points in an outcome domain

b. **Moderate risk of bias:** 2 points in selection domain AND 4 points in comparability domain AND ≥2 points in an outcome domain

c. **High risk of bias:** ≤1 point in selection domain OR ≤3 points in comparability domain OR ≤1 point in an outcome domain

This scoring system is adapted from the Newcastle Ottawa Scale. We gave more weight to Item 5 as these confounding variables have demonstrated a substantial impact on the comparison between RP and RT and overall and cause-specific mortality in prostate cancer research [4].

References

1. Penson D, Albertsen PC, Nelson PS, Barry MSJ. Determining cause of death in prostate cancer: are death certificates valid? J Natl Cancer Inst. 2001; 93(23):1822-3.

2. National Comprehensive Cancer Network. NCCN Clinical Practice Guidelines in Oncology: Prostate Cancer v4.2018. 2018.

3. Loeb S, Smith ND, Roehl KA, Catalona WJ. Intermediate-term potency, continence, and survival outcomes of radical prostatectomy for clinically high-risk or locally advanced prostate cancer. Urology. 2007; 69(6):1170-5.

4. Sooriakumaran P, Nyberg T, Akre O, Haendler L, Heus I, Olsson M, et al. Comparative effectiveness of radical prostatectomy and radiotherapy in prostate cancer: observational study of mortality outcomes. BMJ. 2014; 348:1-13. Available from: http://dx.doi.org/doi:10.1136/bmj.g1502.
